# Dynamic Changes in EEG Power Spectral Densities During NIH-Toolbox Flanker, Dimensional Change Card Sort Test and Episodic Memory Tests in Young Adults

**DOI:** 10.3389/fnhum.2020.00158

**Published:** 2020-05-19

**Authors:** Judith G. Foy, Michael R. Foy

**Affiliations:** Department of Psychology, Loyola Marymount University, Los Angeles, CA, United States

**Keywords:** electroencephalograph, PSD, cognition, attention, memory, NIH-Toolbox

## Abstract

Much is known about electroencephalograph (EEG) patterns during sleep, but until recently, it was difficult to study EEG patterns during conscious, awake behavior. Technological advances such as powerful wireless EEG systems have led to a renewed interest in EEG as a clinical and research tool for studying real-time changes in the brain. We report here the first normative study of EEG activity while healthy young adults completed a series of cognitive tests recently published by the National Institutes of Health Toolbox Cognitive Battery (NIH-TCB), a commonly-used standardized measure of cognition primarily used in clinical populations. In this preliminary study using a wireless EEG system, we examined power spectral density (PSD) in four EEG frequency bands. During baseline and cognitive testing, PSD activity for the lower frequency bands (theta and alpha) was greater, relative to the higher frequency bands (beta and gamma), suggesting participants were relaxed and mentally alert. Alpha, beta and gamma activity was increased during a memory test compared to two other, less demanding executive function tests. Gamma activity was also inversely correlated with performance on the memory test, consistent with the neural efficiency hypothesis which proposes that better cognitive performance may link with lower cortical energy consumption. In summary, our study suggests that cognitive performance is related to the dynamics of EEG activity in a normative young adult population.

## Introduction

The monitoring of brain activity during cognition may help to reveal the dynamics of the working human brain, with versatile non-invasive surface electroencephalography (EEG) being one of the tools in doing so. A comprehensive and validated set of cognitive measurements that can quickly assess brain function across the lifespan, the NIH-Toolbox, is currently available for both research and clinical applications (Gershon et al., 2010). In this study, our goal was to identify specific EEG waveform characteristics occurring during cognition by using this standardized battery of core executive function tasks that included measures of inhibitory control, attention and episodic memory.

Since its discovery by Hans Berger in the 1920’s (Jasper and Carmichael, 1935) in which direct electrical brain activity was recorded from electrodes placed on the scalp, technological advancements in computer systems and brain electrical recording techniques have vastly expanded the ability to examine and understand EEG oscillations related to behavior (see Buzsáki and Draguhn, 2004; Niedermeyer, 2010; Tivadar and Murray, 2019). EEG activity, presumably generated by dendrites of neurons adjacent to the cortical surface of recording electrodes, has been classified into several well-defined neural oscillatory patterns, or frequency bands. The specific EEG frequencies we studied occurred during an eyes-open resting state (baseline), and during a series of cognitive tasks that included the continuous recording of: theta activity (3–7 Hz), alpha activity (8–12 Hz), beta activity (13–29 Hz), and gamma activity (30–40 Hz). Delta activity (1–3 Hz) was not examined, given its primary association with sleep (Steriade et al., 1993; Knyazev, 2007). Changes in EEG frequency bands indicate shifts in firing rate and/or synchronization within cell populations reliably associated with aspects of cognitive processing (Başar et al., 1999; Klimesch, 1999; Ward, 2003; Makeig et al., 2004), and also implicated in various neurological and neuropsychiatric disorders (Engel, 1996; Klimesch, 1999; Griesmayr et al., 2014; Mazzon et al., 2018; Djonlagic et al., 2019). Current platforms to study real-world or simulated environments include those that incorporate relatively simple, inexpensive, rapid setup, wearable, and portable wireless EEG systems whose precision is comparable to laboratory-based wired EEG systems (Debener et al., 2012; De Vos et al., 2014; Cruz-Garza et al., 2017; Ratti et al., 2017). While many benefits of using a portable wireless EEG system include the ability to study cognitive functioning in naturalistic settings and the capability of measuring differences in brain activity in freely moving subjects (Rupp et al., 2018; Blanco et al., 2019; Park and Donaldson, 2019), one limitation to this system compared to large multi-channel traditional EEG systems is that recordings confined to electrode positions covered by the few channels in portable systems precludes extensive multi-channel networks evaluation (Ratti et al., 2017).

The National Institutes of Health Toolbox Cognitive Battery (NIH-TCB) consists of a series of standardized and challenging performance-based cognitive measures specifically developed to “accelerate the pace of discovery in neuroscience research” (National Institutes of Health and Northwestern University, 2006–2017, p. 4). The NIH-TCB has been normed for children, young adults, older adults and the elderly (Gershon et al., 2013). This instrument is a reliable and valuable tool to standardize evaluations in specific populations on cognition, including individuals with intellectual disabilities (Hessl et al., 2016), traumatic brain injury (Tulsky et al., 2017), aging (O’Shea et al., 2016), social anxiety (Troller-Renfree et al., 2015), cognitive impairment in cancer survivors (Apple et al., 2017), and pain (Cruz-Almeida et al., 2019). The cognitive domain battery of this assessment tool includes the evaluation of higher-level brain functions such as thinking, judging and remembering. While the above studies focused on clinical populations, an examination of EEG activity both during baseline (resting state: a period of time when participants are assigned no cognitive task) and while healthy participants perform these standardized cognitive tasks has yet to be reported. Here, we used three independent measures of cognitive function in healthy, young adults: inhibition control and attention (Flanker Inhibitory Control and Attention Test), cognitive flexibility (Dimensional Change Card Sort Test), and episodic memory (Picture Sequence Memory Test) (Weintraub et al., 2014). Several recent studies have examined whether performance on the NIH-TCB can be predicted by EEG activity during baseline measure only (Brucar et al., 2019; Požar et al., 2020). An understanding of normative EEG patterns is needed given the potential research and clinical applications of EEG *during* human cognition.

Wireless EEG systems are powerful tools for studying everyday cognition in normal and clinical populations and to investigate the effects of various interventions on behavior. The current study is the first to examine neural activity in normal young adults during a powerful, commonly used standardized cognitive assessment tool, the NIH-TCB. As a first step, this brief report examines single-trial power spectral density (PSD) EEG activity in four frequency bands (theta, alpha, beta, gamma) measured during baseline and during three cognitive tests. We hypothesize that alpha PSD will decrease, whereas beta, theta, and gamma PSD will increase in the cognitive tasks relative to baseline. This study also describes links between these EEG measures and performance on these cognitive tests. We hypothesize that alpha PSD will be inversely correlated whereas beta, theta and gamma PSD will be positively correlated with performance in these cognitive tasks.

## Materials and Methods

### Participants

The final sample consisted of 29 undergraduate subject pool volunteers (19 female, 28 right-handed), aged 18–22 years (*M* = 19.0, *SD* = 1.1 years) who were native English speakers. Selected volunteers were recruited from a pool of approximately 300 students, and none of the volunteers were current or former students of the faculty members conducting the study at the time of their participation. The data from three participants were corrupted with excessive artifacts during data collection, and were not used. Participants who earned partial course credit were treated in accordance with the APA Code of Ethics (American Psychological Association, 2017). All subjects had normal or corrected-to-normal vision, and through a survey conducted prior to the study, self-reported no neurological, anxiety, depressive, sleep or cardiovascular disorders, and no prior experience with the cognitive measures used in this study. Participants abstained from alcoholic beverages 24 h, caffeinated beverages 12 h, and nicotine 1 h prior to the study.

### Cognitive Measures

An iPad app version of the NIH-TCB that normalizes scores for age, sex, education and ethnicity was used during three separate cognitive measures: the Flanker Test, the Dimensional Change Card Sort (DCCS) Test, and the Picture Sequence Memory (PSM) Test (Weintraub et al., 2013). For all NIH-TCB measures, instructions were provided visually and orally through the iPad; participants made their selections on the iPad with their dominant hand. Practice and test phases (4 min for the Flanker and DCCS tests; 8 min for the PSM test) were given to each participant. A scoring algorithm for each test integrates accuracy and reaction time (RT) yielding computed age-adjusted scores, reflecting relative performance based on a nationally representative normative sample (*M* = 100, *SD* = 15) within the same age band yield. For the young adults in this study, the age band used in the norming studies for the NIHTB is 18–29 years (Slotkin et al., 2012). Accuracy per item type was calculated as the mean number of items answered correctly. All RTs for correct items are reported in seconds from a home base position to making the item response.

#### Flanker Test

The NIH-TCB version of the Eriksen flanker test is derived from the Attention Network Test (Rueda et al., 2004), an executive function measure of inhibitory control and attention (Weintraub et al., 2013). The Flanker test requires participants to focus on a visual target while ignoring stimuli on either side (i.e., flanking) the target and to choose one of two buttons on the screen that corresponds to the direction in which the middle arrow was pointing. On 12 congruent trials, all arrows point in the same direction. On 8 incongruent trials, the flanking arrows point in the opposite direction of the middle arrow. Congruent and incongruent trials are mixed and standardized for each participant. Congruent and incongruent scores and RT for the behavioral assessment were calculated as the mean accuracy and RT on each of the congruent and incongruent trials, respectively. Validation of the computerized version of this test has a test-retest reliability of 0.85, and intra-class correlations of 0.83 (95% CI: 0.74–0.89) (Zelazo et al., 2014).

#### Dimensional Change Card Sort (DCCS) Test

The DCCS test (Zelazo et al., 2014), similar to the Wisconsin Card Sorting Task (Milner and Petrides, 1984), is an executive function measure of cognitive flexibility (Weintraub et al., 2013). In the NIH-TCB version, following instructions and a practice phase, participants match a series of two test pictures located at the bottom of the screen (e.g., a yellow ball and a blue truck) depending on a word cue (e.g., “color”) located in the top half of the screen according to one dimension (in this test, shape). Twenty-three trials are designated as “repeat” trials. Seven “switch” trials are also employed, in which the participant must change the dimension being matched from preceding trials (to “color”).^1^ In a validation of the computerized version of this study, Zelazo and colleagues reported test-retest reliability of 0.85 and intra-class correlations of 0.81 (95% CI: 72–87).

#### Picture Sequence Memory Test

The PSM test is a measure of episodic memory, and involves recalling two series of illustrated objects and activities that are presented in a standardized order arrayed in a large square on the iPad screen, with corresponding audio-recorded phrases played through the iPad. Participants are asked to recall the sequence of pictures by moving the images with the forefinger on their dominant hand of the touchscreen iPad to the locations of small blank squares positioned around the central box in the order in which they recalled them. The practice phase consists of four items. The first learning trial during the test phase consists of 15 pictures, whereas the second trial consists of 18 pictures. The accuracy score for this test consists of credit given for each adjacent pair of pictures correctly placed in the large square in the center of the screen up to the maximum value for each trial (14 for trial 1, 17 for trial 2). RT for each trial is calculated from the time that the participant has been shown all pictures in that trial to the participant indicating that all the pictures have been placed into the squares along the side of the screen. Test-retest reliability of 0.84 and intra-class correlations of 0.77 of the computerized version of this test have been reported (Dikmen et al., 2014).

### Electroencephalograph (EEG) Measures

Electrophysiological signals were recorded from a B-Alert Live X10 wireless Bluetooth (Advanced Brain Monitoring (ABM), Inc., Carlsberg, CA, United States) wireless EEG sensor headset. Nine light-weight electrodes on a sensor strip, referenced to linked mastoids, were used to collect EEGs from participants at a sampling rate of 256 Hz, along with an additional channel that recorded electrocardiogram activity. The sensors were placed over frontal, central, and parietal regions according to the International 10–20 system coordinates (sensor sites: Fz, F3, F4, Cz, C3, C4, POz, P3, P4). Amplification, digitization and radio-frequency transmission of the signals was accomplished with miniaturized electronics in the portable unit worn on the head.

ABM algorithms identified and decontaminated 3 or more data point spikes with amplitudes greater than 40 mV associated with excessive muscle activity, eye blinks (fast and slow), excursions due to movement artifacts, amplifier saturations, and spikes. Following filtering and artifact removal, Power Spectral Densities (PSD) were computed by performing Fast Fourier Transform for theta (3–7 Hz), alpha (8–12 Hz), beta (13–29 Hz), and gamma (30–40 Hz) frequency bands.

#### Baseline EEG Measurement

Immediately prior to administration of the 3 NIH-TCB tests, participants were instructed to remain still and relaxed for 2 min eyes closed, and 2 min eyes open rest-periods. The latter was chosen as the baseline period in this study.

#### Power Spectral Density (PSD)

For each participant, continuous EEG activity was recorded until the completion of the entire testing session. Electronic markers were placed in the EEG record to indicate the beginning and end of the rest periods, practice and test phases. Data were segmented offline into discrete, individualized periods for each subject consisting of baseline, instructions and practice, and cognitive tests associated with each cognitive task. Only the baseline and test data are included in the current analyses. For PSD computation, each window size was one epoch containing one second of data (i.e., 256 decontaminated EEG samples). A 50% overlapping Kaiser window was applied to smooth the PSD data over 3 windows. For the current analyses, PSD was averaged across electrode sites.

## Results

All statistical analyses were conducted using the Statistical Package of Social Sciences 24 (SPSS). Prior to performing inferential statistics, the data were examined for normality by two methods: skewness and kurtosis were within acceptable limits (±2) according to George and Mallery (2010) and the Q-Q plots were consistent with normal distributions (Thode, 2002, p. 21). The repeated measures Analyses of Variance (ANOVAs) included Mauchly’s test of sphericity. Where significant violations of the assumption of homogeneity of variance were found, Greenhouse-Geisser adjustments were made to the df in the analyses (where *χ*^2^ was significant, all *p* ≤ 0.0001). Follow-up analyses after significant main and interaction effects for both between- and within ANOVAs always used Bonferroni-corrections to counteract the problem of multiple comparisons.

### Cognitive Measures (NIH Toolbox: NIH-TCB)

**FIGURE 1:**
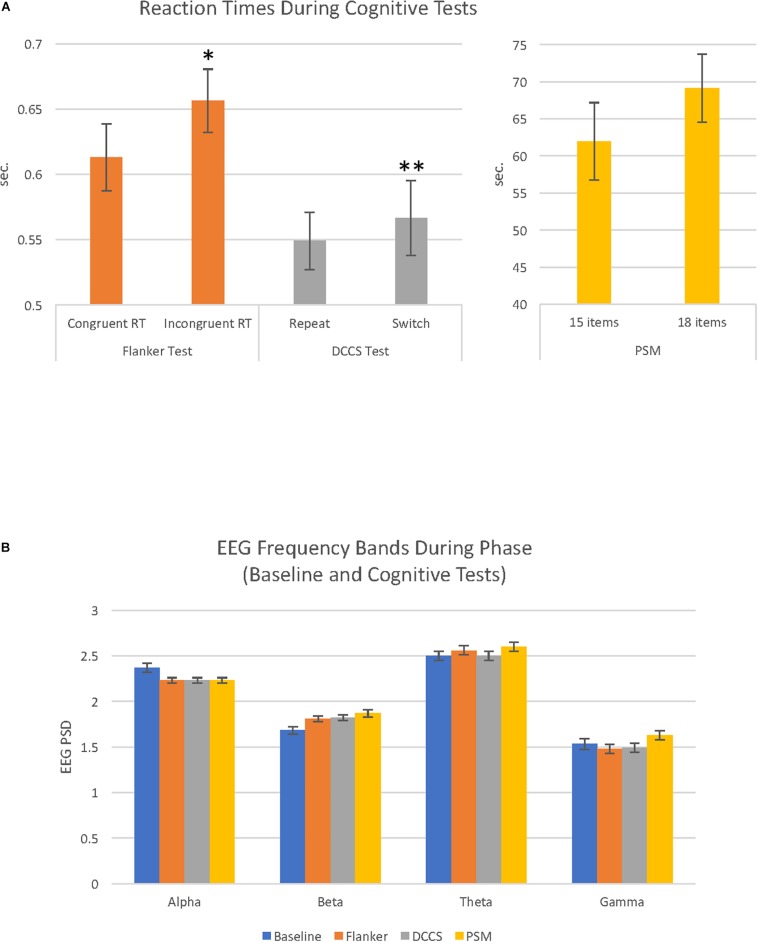
Cognitive test reaction times and EEG activity recorded during baseline and cognitive test conditions. **(A)** Bar graphs illustrate reaction times (RT) during the 3 cognitive tests (Flanker, DCCS and PSM). Each cognitive test had 2 conditions that were evaluated in paired-samples *t*-tests; for the Flanker test, congruent reaction time (RT) vs. incongruent RT; for the DCCS test, repeat vs. switch; for the PSM test, trial 1 (15 items) vs. trial 2 (18 items). These comparisons were significantly different in the Flanker (^∗^*p* < 0.05) and DCCS (^∗∗^*p* < 0.01). **(B)** bar graphs illustrate EEG power spectral density (PSD) for alpha, beta, theta and gamma EEG frequency bands recorded during baseline (eyes open resting state), and the 3 cognitive tests (Flanker, DCCS, PSM). Alpha and theta PSD were higher compared to beta and gamma during the cognitive tests and baseline (all comparisons *p* = 0.0001). For details of other significant differences (see Table 1).

Within each cognitive test, two categories of trials were compared with paired samples *t*-tests. During the Flanker test, participants performed significantly faster on the congruent than incongruent trials, *t*(28) = 2.67, *p* = 0.013. During the DCCS test, participants had faster RTs to repeat than to switch trials, *t*(28) = 3.01, *p* = 0.005. For the PSM test, scores for the first trial were higher than for the second trial which had more items, *t*(28) = 5.88, *p* = 0.0001, and the RT was longer for the second trial than for the first trial, though the result did not reach statistical significance, *t*(28) = 1.85, *p* > 0.05 (Figure 1A). In order to investigate the possibility of a speed-accuracy tradeoff (Zimmerman, 2011), RTs and raw scores were correlated. In only the DCCS was there evidence of a speed-accuracy tradeoff, *r*(29) = 0.44, *p* = 0.01 (Flanker: *p* > 0.05; PSM: *r* = −0.62, *p* = 0.001). The scores and RTs used in the remainder of the analyses are shown in Supplementary Table S1.

### EEG Power Spectral Density (PSD)

A 4 (phase: baseline, flanker, DCCS, PSM) × 4 (frequency band: alpha, beta, theta, gamma) repeated measures ANOVA revealed a main effect of phase, *F*(2.85, 79.87) = 15.38, *MSE* = 0.02, *p* = 0.0001, η*_*p*_*^2^ = 0.35 (Figure 1B). There was also a main effect of frequency band, *F*(1.70, 47.65) = 160.54, *MSE* = 0.26, *p* = 0.0001, η*_*p*_*^2^ = 0.85. For EEG frequency band PSD magnitudes, theta was largest, followed by alpha, then beta and gamma PSD showing the smallest amplitudes. All comparisons were significant at *p* = 0.0001. Average EEG PSD values for each frequency band during baseline and the cognitive tests are shown in Table 1.

**TABLE 1:** Descriptive statistics for EEG PSD and summary of ANOVA results and *post hoc* testing.

EEG measure	Resting	Flanker	DCCS	PSM	*F*	*p*	η*_*p*_*^2^
Alpha	2.37 (0.05)	2.23 (0.03)^a^	2.24 (0.03)^a^	2.23 (0.03)^a^	13.58	0.0001	0.33
Beta	1.68 (0.04)	1.81 (0.03)^a^	1.82 (0.03)^a^	1.84 (0.04)^a^	16.68	0.0001	0.37
Theta	2.50 (0.05)	2.56 (0.05)^a^	2.50 (0.05)^b^	2.60 (0.05)^a^	14.31	0.0001	0.34
Gamma	1.53 (0.06)	1.48 (0.05)	1.49 (0.05)	1.63 (0.05)^c^	8.99	0.001	0.22

**The standard error of the mean is in parentheses. Significance (p) reflects Greenhouse-Geisser corrections to degrees of freedom due to significant violations of Mauchly’s test of sphericity. ^*a*^Significantly different from baseline. ^*b*^Significantly different from Flanker and from PSM. ^*c*^Significantly different from Flanker and DCCS.**

A significant interaction between phase and frequency band, *F*(3.22, 90.10) = 16.27, *MSE* = 0.024, *p* = 0.0001, η*_*p*_*^2^ = 0.37, was followed up with separate one-way repeated measures ANOVAs for each phase and frequency band, described below.

During baseline, there was a significant effect of frequency band activity, *F*(1.67, 46.85) = 111.07, *MSE* = 0.11, *p* = 0.0001, η*_*p*_*^2^ = 51. As shown in Figure 1B (Table 1), the highest PSD magnitude during baseline was in the theta frequency band. Theta and alpha band activity was significantly larger than gamma PSD. Beta was significantly smaller than theta and significantly larger than gamma PSD (all *p* = 0.0001). Alpha and theta PSD magnitudes were not significantly different from each other (*p* = 0.059).

### EEG Measures During Cognitive Tests

A one-way ANOVA revealed a significant effect of phase on alpha PSD, *F*(1.58, 44.32) = 13.58, *MSE* = 0.018, *p* = 0.0001, η*_*p*_*^2^ = 0.33. Alpha PSD was largest during baseline compared to alpha during the Flanker (*p* = 0.003), DCCS (*p* = 0.001), and PSM tests (*p* = 0.002), but there were no differences between alpha during the cognitive tests Figure 1B (Table 1).

There was a significant effect of phase on beta PSD, *F*(1.85, 51.75) = 16.68, *MSE* = 0.02, *p* = 0.0001, η*_*p*_*^2^ = 0.37. Beta PSD was smaller during the baseline than during the Flanker (*p* = 0.001), DCCS (*p* = 0.001), and PSM tests (*p* = 0.001). There were no significant PSD differences between the tests for beta Figure 1B (Table 1).

There was a significant effect of phase on theta PSD, *F*(2.53, 70.81) = 14.31, *MSE* = 0.006, *p* = 0.0001, η*_*p*_*^2^ = 0.34. Theta PSD was larger during the Flanker (*p* = 0.035) and PSM (*p* = 0.001) tests than during the baseline. Theta PSD during the DCCS test was smaller than during the Flanker and PSM tests (both *p* = 0.0001), but was not significantly different from the baseline period Figure 1B (Table 1).

There was a significant effect of phase on gamma PSD, *F*(2.26, 63.40) = 7.76, *MSE* = 0.025, *p* = 0.001, η*_*p*_*^2^ = 0.22. Although the increase in gamma from baseline to the cognitive tests did not reach statistical significance, gamma PSD was larger during the PSM test than during the two executive function tests Figure 1B (Table 1): Flanker (*p* = 0.001) and DCCS tests (*p* = 0.002).

### Correlations Between EEG and Performance

**FIGURE 2:**
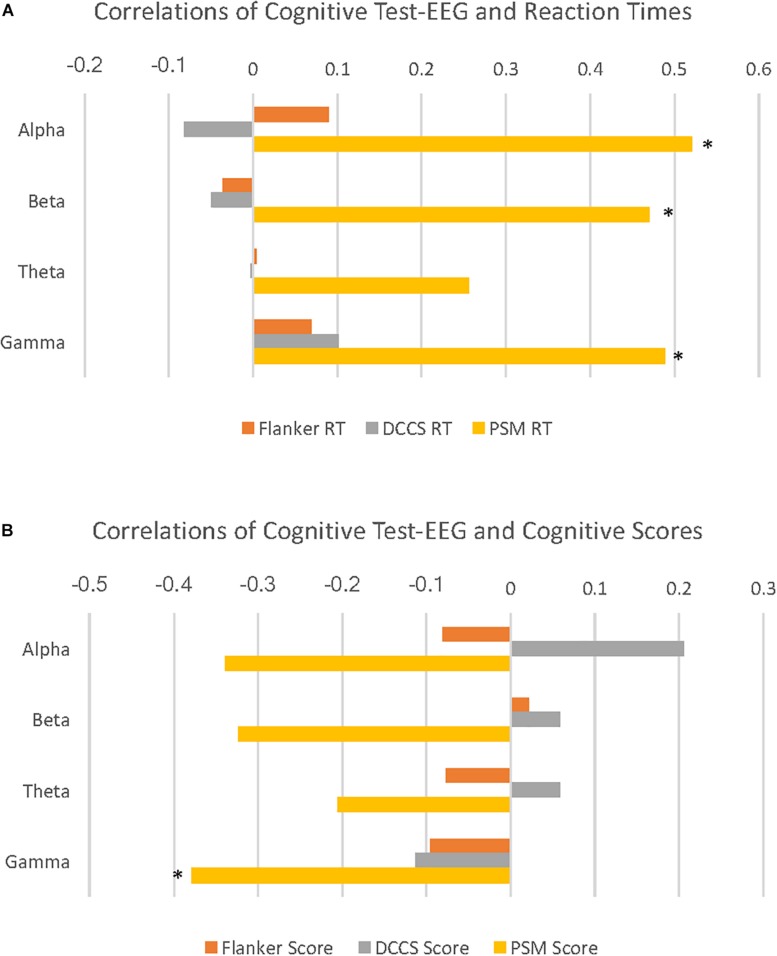
Cognitive test-EEG correlations with reaction times and age-corrected standard scores on the cognitive tests. **(A)** horizontal bar graphs illustrate correlations of PSD for 4 EEG frequency bands with reaction times (RT) during 3 cognitive tests. Here, alpha, beta and gamma EEG PSD activity are all significantly positively correlated with Picture Sequence Memory (PSM) RT. **(B)** horizontal bar graphs illustrate correlations of 4 EEG frequency band PSD with age-corrected standard scores (cognitive scores) during 3 cognitive tests. Here, gamma activity is significantly negatively correlated with PSM scores. (^∗^*p* < 0.05).

**FIGURE 3:**
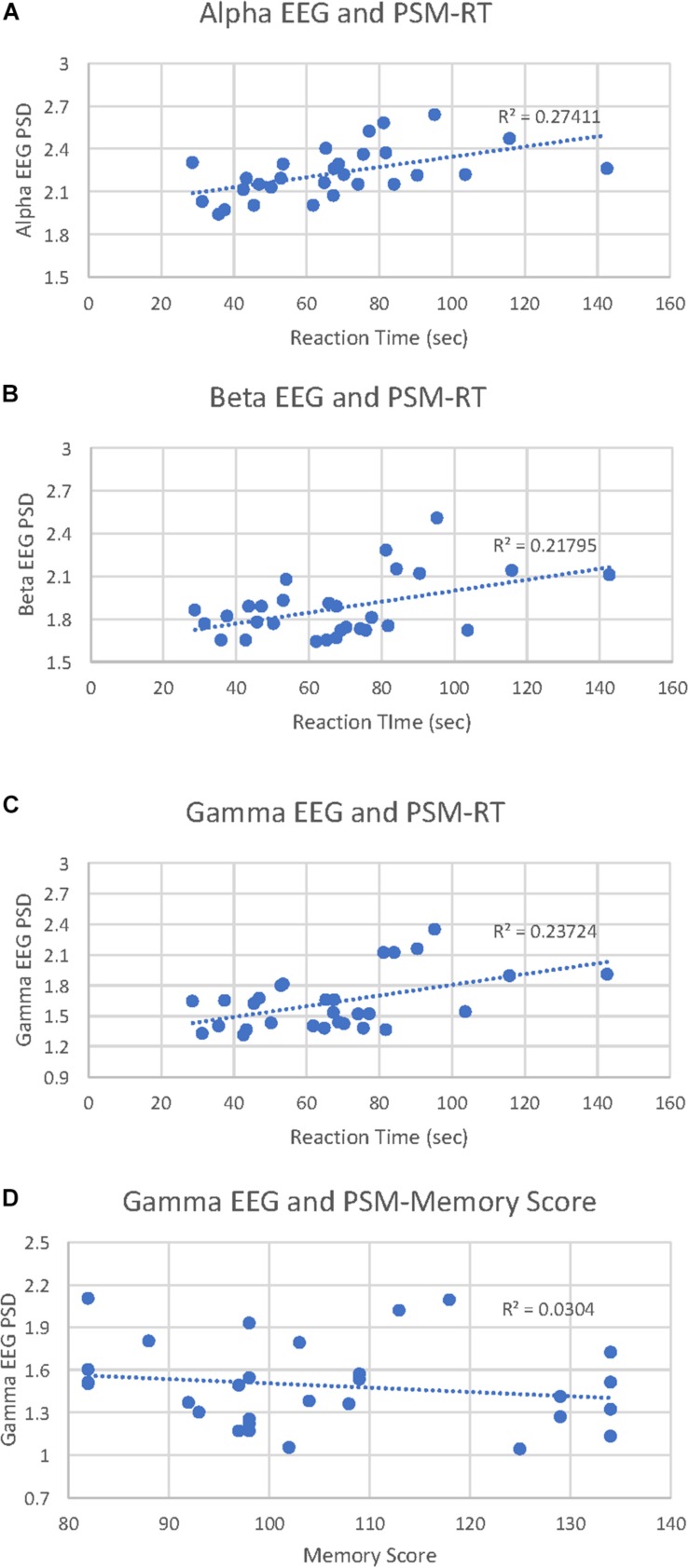
Picture Sequence Memory (PSM)-EEG activity correlations with PSM reaction times and cognitive scores. **(A)** scatterplot displays all data points for the PSM-alpha EEG PSD correlation with PSM-reaction time (RT). **(B)** scatterplot displays all data points for the PSM-beta EEG PSD correlation with PSM-RT. **(C)** scatterplot displays all data points for the PSM-gamma EEG PSD correlation with PSM-RT. **(D)** scatterplot displays all data points for the PSM-gamma EEG PSD correlation with PSM-cognitive score.

PSM-alpha, *r*(29) = 0.52, *p* = 0.004, PSM-beta, *r*(29) = 0.47, *p* = 0.01, and PSM-gamma, *r*(29) = 0.49, *p* = 0.007 were significantly correlated with PSM-RT (Figure 2A). PSM-gamma was also inversely correlated with the PSM-score, *r*(29) = -0.38, *p* = 0.04 (Figure 2B). Thus, faster PSM-RTs were associated with lower alpha, beta and gamma PSD during this memory test; higher scores were linked with lower gamma PSD. Scatterplots of significant correlations between EEG measures and cognitive performance measures are displayed in Figures 3A–D. Baseline, Flanker-EEG, and DCCS-EEG PSDs were not significantly correlated with performance measures. See Supplementary Table S2 for correlations between all baseline and cognitive test-EEG measures.

## Discussion

To contribute to the normative literature on the relationship between EEG activity and cognition, we compared EEG data collected from healthy young adults *during* NIH-TCB tasks to a baseline (eyes open rest-period) condition. We also explored the relationship between EEG PSD and performance.

### Cognitive Performance

Our behavioral results replicated previous findings for each of the cognitive measures. In the Flanker test, a measure of inhibitory control (Zelazo et al., 2014), an executive function (Miyake et al., 2000), we found longer RTs for incongruent than for congruent trials indicating that the test successfully manipulated demands on inhibitory control (Eriksen and Eriksen, 1974). Impaired speed on incongruent trials is thought to reflect the additional processing time needed to override motor plans and maintain adequate accuracy (Eriksen, 1995). RT and accuracy data were inversely related in the Flanker test indicating that there were no speed-accuracy tradeoffs in our study.

The DCCS is a measure of cognitive flexibility (Weintraub et al., 2013), also an executive function (Miyake et al., 2000). It is similar, though simpler, to the Wisconsin Card Sorting Task which activates various brain regions thought to involve working memory (Berman et al., 1995). As expected, switch trials were associated with significantly longer RTs in our study compared to repeat trials reflecting attentional inertia for the first-learned dimension (Diamond and Kirkham, 2005; Ezekiel et al., 2013). Longer RTs during the DCCS are associated with greater accuracy suggesting that participants adopted a strategy of maximizing accuracy by slowing responses (Zimmerman, 2011).

The PSM test is a measure of episodic memory (Dikmen et al., 2014). We found that reaction times were longest on this task compared to the other cognitive tasks (more items per trial), and for the second trial, which consisted of more items than for the first trial. On this test, an inverse relationship between RT and accuracy suggests that subjects did *not* adopt a strategy indicative of a speed-accuracy tradeoff during this task.

### Baseline EEG

During baseline, PSDs for the lower frequency bands (theta and alpha) were greater relative to the higher frequency bands (beta and gamma). Theta activity may reflect activity in the default mode network (Scheeringa et al., 2008), while high alpha activity is typical during low arousal resting states (Barry et al., 2007). The high PSD values for theta and alpha relative to the other frequency bands show that the participants in the current study were relaxed but mentally alert (Lafrance and Dumont, 2000) during the baseline task. Our results add to the literature suggesting the brain at rest is an active brain (Damoiseaux et al., 2006; Bonnard et al., 2016). No RS-EEG measures predicted cognitive performance. In a forthcoming study, we will explore both baseline PSD and NIH-TCB cognitive test PSD patterns by electrode position.

### Cognitive Test EEG

The lower frequency bands, theta and alpha, were maximal relative to the higher frequency bands during the baseline period and during the cognitive tests, demonstrating the predominance of these frequency bands and suggesting that our participants were relaxed and mentally alert (Lafrance and Dumont, 2000; Barry et al., 2007; Scheeringa et al., 2008) throughout the study. Alpha desynchronization was apparent in all three cognitive tests relative to the baseline, consistent with a large body of literature suggesting that alpha activity may be an attentional suppression mechanism when dimensions of stimuli need to be ignored (for reviews see Foxe and Snyder, 2011; Klimesch, 2012).

Theta, beta, and gamma PSD increased in at least one cognitive test compared to baseline. Theta band activity predominated in baseline, as well as during the cognitive tasks relative to the other frequency bands, reflecting the attentional (Ishii et al., 1999), control (Sauseng et al., 2010; Reinhart et al., 2015) and memory demands (Klimesch, 1999; Gevins and Smith, 2000; Jensen and Tesche, 2002; Hanslmayr et al., 2008) of this study. Relative to baseline, theta PSD increased in the Flanker and PSM tests, consistent with other studies linking theta with focused attention, controlled processing, and memory. An intriguing finding was that theta band activity was not significantly different from baseline in the DCCS test, reflecting theta band desynchronization relative to the preceding Flanker test. Theta band desynchronization has been associated with alertness increases (Lafrance and Dumont, 2000) and to stress (Gärtner et al., 2015). The DCCS test may have been more challenging for our participants compared to the preceding Flanker test.

Synchronization in beta band activity occurred during each of the cognitive tests relative to baseline. Given that participants in the present study used the same response set throughout the study (movements on an iPad touch-screen for target stimuli), these results are consistent with the view that beta band activity reflects maintenance of a sensori-motor set (Engel and Fries, 2010).

In agreement with other studies reporting gamma band activity linked with working memory (Roux et al., 2012; Roux and Uhlhaas, 2014) and increased cognitive load (Whittier et al., 2019), we found that gamma activity was larger during the PSM test than during the other two executive function tests (Flanker and DCCS). Applicable to the PSM test, gamma synchronization is also associated with a change in the stimulus set and increased proactive control (Engel and Fries, 2010; Boudewyn et al., 2019).

### Correlation Between EEG Measures and Cognitive Performance

Alpha, beta, and gamma (PSM) were significantly correlated with performance. Neither baseline-EEG measures nor theta activity during any cognitive tests was related to performance.

Alpha, beta and gamma desynchronization predicted better performance on the PSM, a measure of episodic memory (Dikmen et al., 2014). Lower PSD in the alpha and beta bands has previously been linked to better memory performance (Zhu et al., 2019). To our knowledge, we are the first to report that gamma desynchronization is also linked to better memory performance: this effect was robust in that lower gamma PSD was linked with both scores and RT (faster), whereas lower alpha and beta PSD were only linked with RT (also faster). These findings of desynchronization in alpha, beta and gamma bands are consistent with the neural efficiency hypothesis: better cognitive performance is sometimes associated with lower cortical energy consumption (Genç et al., 2018, 2019; Ramchandran et al., 2019).

In conclusion, to our knowledge, the present study is the first to characterize EEG and cognitive data obtained while healthy young adults completed tasks from the NIH-TCB, as well as during rest, and linking the findings to performance. This descriptive report provides a starting point for future studies to examine task/test relevant EEG activity in populations differing along important dimensions such as age or clinical diagnosis, and the effect of drugs or behavioral interventions. Just as RS-EEG measures are now well-recognized as important associates of cognitive outcomes (Buzsáki and Draguhn, 2004), systematic examination of EEG measures *during* standardized NIH-TCB testing could help us to further understand cognitive performance and EEG recording in a variety of diverse populations.

## Data Availability Statement

The raw data supporting the conclusions of this article will be made available by the authors, without undue reservation, to any qualified researcher.

## Ethics Statement

The studies involving human participants were reviewed and approved by Loyola Marymount University Institutional Review Board. All participants provided their written informed consent to participate in this study.

## Author Contributions

Both authors contributed equally to the planning and execution of this study, analysis of the data, and manuscript preparation.

## Conflict of Interest

The authors declare that the research was conducted in the absence of any commercial or financial relationships that could be construed as a potential conflict of interest.

## Footnotes

1 NIH-TCB designation of “repeat” trials includes trials immediately following “switch” trials, possibly confounding this measure. For the behavior assessment (3.1.1) we used only the 7 items immediately preceding switch items and following the same item as “repeat” trials.
