# Supplementary material for: Dynamic Changes in EEG Power Spectral Densities During NIH-Toolbox Flanker, Dimensional Change Card Sort Test and Episodic Memory Tests in Young Adults
Source: Front Hum Neurosci. 2020 May 19;14:158. doi: 10.3389/fnhum.2020.00158 (PMC7248326; doi:10.3389/fnhum.2020.00158)
Supplement: Supplementary file 1 [file Table_1.DOCX]

**Supplementary Material**

**Table 1** Descriptive statistics (mean and standard deviation: SD) for performance (score and reaction time: RT) during cognitive tests

**______________________________________________**

**Measure Mean SD Range**

**______________________________________________**

Flanker Score 84.19 12.74 58 - 107

DCCS Score 99.81^1^ 15.78 69 - 130

PSM Score 106.52^2^ 16.70 82 – 134

______________________________________________

Flanker RT 0.64 0.13 0.41 - 0.89

DCCS RT 0.56^1^ 0.13 0.30 - 0.84

PSM RT 65.55^1,2^ 25.58 28.71 - 142.67

***_______________________________________________***

***Score:*** *A one-way ANOVA (cognitive test: Flanker, DCCS, PSM) was conducted for the age-corrected scores. There was a significant main effect of cognitive test on score, F(1.32, 39.68) = 21.46, MSE = 286.52, p = .001, , η_p_^2^ = .42, using Greenhouse-Geisser-corrected df. 1: significantly different from Flanker scores, p = .0001 (Bonferroni-test). 2: significantly different than the Flanker and DCCS score, p = .0001 (Bonferroni-test).*

***RT:*** *A one-way ANOVA (cognitive test: Flanker, DCCS, PSM) was conducted for RT. There was a significant main effect of cognitive test on RT, F(1,28) =192.02, MSE = 451.51, p = .0001η_p_^2^ = .87, using Greenhouse-Geisser-corrected df.*

*1: significantly different from Flanker RT, p = .0001 (Bonferroni-test). 2: significantly different from the Flanker and DCCS RT, p = .0001 (Bonferroni-test).*

**Table 2** Correlations between EEG measures and performance (score and reaction time (RT) during cognitive tests

**__________________________________________________________________**

**Measure Flanker Flanker DCCS DCCS PSM PSM**

**Score RT Score RT Score RT**

**__________________________________________________________________**

**Baseline**

Alpha .03 -.06 .28 -.19 -.13 .27

Beta -.07 .01 -.18 .16 -.02 .09

Theta -.26 .17 -.14 .21 -.27 .29

Gamma -.10 .02 -.24 .21 -.02 .08

__________________________________________________________________

**Flanker**

Alpha -.08 .09 - - - -

Beta .02 -.04 - - - -

Theta -.08 .01 - - - -

Gamma -.10 .07 - - - -

__________________________________________________________________

**DCCS**

Alpha - - .21 -.08 - -

Beta - - .06 -.05 - -

Theta - - .06 -.00 - -

Gamma - - -.11 .10 - -

__________________________________________________________________

**PSM**

Alpha - - - - -.34 **.52****

Beta - - - - -.32 **.47***

Theta - - - - -.21 .26

Gamma - - - - **-.38* .49****

__________________________________________________________________

*Table 2 displays Pearson correlations between baseline and performance on all cognitive tests, and between EEG correlations for each cognitive test and performance on the respective cognitive test. Bold text indicates significant correlations, *: p < .05, **: p < .01.*
